# A versatile survey method for repeatedly monitoring individual road-kill

**DOI:** 10.7717/peerj.21636

**Published:** 2026-09-21

**Authors:** Jon J. Sullivan, Giorgia Vattiato, Chris N. Niebuhr

**Affiliations:** 1 Department of Pest-Management and Conservation, Lincoln University, Lincoln, New Zealand; 2 Manaaki Whenua–Landcare Research, Lincoln, New Zealand

**Keywords:** Roadkill, Wildlife, Ecological monitoring, Birds, Mammals, Wildlife vehicle collision, New Zealand, Road ecology

## Abstract

Road-kill is increasingly recognised as an important source of mortality for wildlife, especially in densely populated urban and rural landscapes. Monitoring road-kill on fine spatial and temporal scales is necessary to better understand how the design of road networks affects road-kill. We report on a practical method for consistently and repeatedly surveying road-kill. This includes geotagging individual carcasses, reporting where on or by the road each carcass is found, categorising the age and state of each carcass, and noting whether each carcass is new or previously reported. All this can practically be done with a smartphone while on foot, biking, or as a passenger in a motor vehicle. Repeatedly mapping all carcasses, whether or not they have previously been reported, allows for estimates of carcass persistence and detection probability for both taxa and road conditions. We demonstrate the effectiveness of the method by reporting on its frequent use along one 16.2 km route of urban and rural roads in New Zealand over 12 years. Over this period, on 1,652 surveys, 4,034 new road-kill carcasses were observed, 62.4% of which were birds, 31.1% were mammals, and 3% were butterflies. Using carcass age and persistence, we estimate that the road-kill rate along this route has been at least 3,760–6,093 road-kill/100 km/year. There was considerable variation among taxa in carcass persistence and carcass position on and by the road, both of which will introduce biases into road-kill estimates if not accounted for. To better understand road-kill and estimate road-kill rates, we encourage road-kill studies to geotag all individual carcasses and track their persistence.

## Introduction

Vehicle collisions result in several hundred million bird mortalities worldwide each year, including estimates of 14 million in Canada, 27 million in England, and 80–340 million in USA (as cited in Husby, 2017). In New Zealand, much of the reported road-kill is of smaller, introduced mammals, *e.g.*, common brushtail possums (*Trichosurus vulpecula*), hedgehogs (*Erinaceus europaeus*), European rabbits (*Oryctolagus cuniculus*) and hares (*Lepus europaeus*), as opposed to ungulates, resulting in little public concern regarding risk to motorists. Limited information exists on the impacts of vehicle-caused mortality on avian species or populations in New Zealand, although some reports exist that provide evidence of birds being killed by vehicles and highlight risks to populations and individuals (*e.g.*, Beauchamp, 2009; Brockie, Sadleir & Linklater, 2009; Sadleir & Linklater, 2016; Flux, Tryjanowski & Zduniak, 2022). We have only identified eight studies within the peer-reviewed scientific literature that are primarily dedicated to road-kill in New Zealand. For these studies (which are discussed in detail below), reporting, survey methods, sampling designs, and analyses differed, making simple comparisons difficult.

Direct comparisons with the international literature may be even more difficult, due to differences in New Zealand avifauna. For example, some of New Zealand’s endemic bird species have evolved flightlessness in the historical absence of mammalian predators, likely increasing their vulnerability to road mortality. Furthermore, New Zealand studies were typically not conducted within major habitats of highly threatened species, and thus present data highly dominated by more common native and introduced species (*e.g.*, Sadleir & Linklater, 2016) conducted surveys in peri-urban and farmland habitats). Currently 80 bird species in New Zealand are classified ‘Threatened’ and 98 as “At Risk” by the NZ Threat Classification system used by the Department of Conservation (Robertson et al., 2021).

Mitigation efforts are designed to reduce mortality/injury due to road-kill. Over 40 types of road mitigation, designed to reduce road-kill of wildlife, exist globally, and these are discussed in detail in various reviews and reports (*e.g.*, Huijser et al., 2008; Van Der Ree, Smith & Grilo, 2015; Rytwinski et al., 2016). There is no ‘one size fits all’ mitigation practice, and each site and/or species scenario must be individually considered. Regardless of the type of mitigation used, proper evaluation of the effectiveness of any effort should include road-kill data collected prior to implementation to allow comparisons to data collected after the mitigation attempts have taken place. In order to collect such data, an appropriate sampling method must be available that accurately estimates road-kill numbers while minimising and quantifying any biases.

Multiple methods have been used to monitor road-kill of a given species or in a certain area (Van Der Ree, Smith & Grilo, 2015). Some monitoring is passive (often opportunistic and without an experimental design), such as police or road agency reports and road-kill citizen science efforts. Other monitoring is active (implementing a predetermined, experimental design), such as government mandated surveys (often poorly designed and under-resourced) and research-based surveys (question-driven and usually more rigorous) (Van Der Ree, Smith & Grilo, 2015). Regardless of the type of survey method used, there is often bias associated with the data collected, such as overrepresentation of certain species or underestimations of true road-kill numbers occurring in the landscape, which makes comparing data difficult (Guinard, Julliard & Barbraud, 2012). Unbiased estimates of road-kill abundance are necessary to accurately assess the impacts on a population, community, or species, as well as measure the effectiveness of any mitigation measures designed to reduce these impacts. One review reported that out of 645 publications discussing research issues related to wildlife vehicle collisions, only 7% dealt with the approach of data collection (Pagany, 2020). Guinard, Prodon & Barbraud (2015) suggested that to reduce bias in road-kill surveys, three parameters need to be quantified:

– Persistence (the probability that the carcass was not removed from the road between counts; or the inverse of carcass disappearance)
– Entry (the probability that a new carcass appears on the road between counts)
– Detectability (the probability of detecting the carcass).

Very few peer-reviewed studies have reported on road-kill of birds in New Zealand, with even fewer having conducted designed sampling. Of the eight New Zealand studies with road-kill as the primary focus (Burger & Gochfeld, 1992; Brockie, 2007; Washington et al., 2008; Beauchamp, 2009; Brockie, Sadleir & Linklater, 2009; Freeman, 2010; Sadleir & Linklater, 2016; Flux, 2019), only five focussed on multiple species. Seven studies conducted driving counts, with one conducting cycling counts.

Here we present a versatile, consistent method to ascertain rates of road-kill that has been applied over the past two decades, mostly while travelling by bike, in part of New Zealand. We demonstrate that it is practical to track individual road-kill carcasses on daily work-commutes over extended periods of time with relatively little effort, in a way that allows road-kill detection and persistence biases to be estimated.

## Materials & Methods

**Figure 1:**
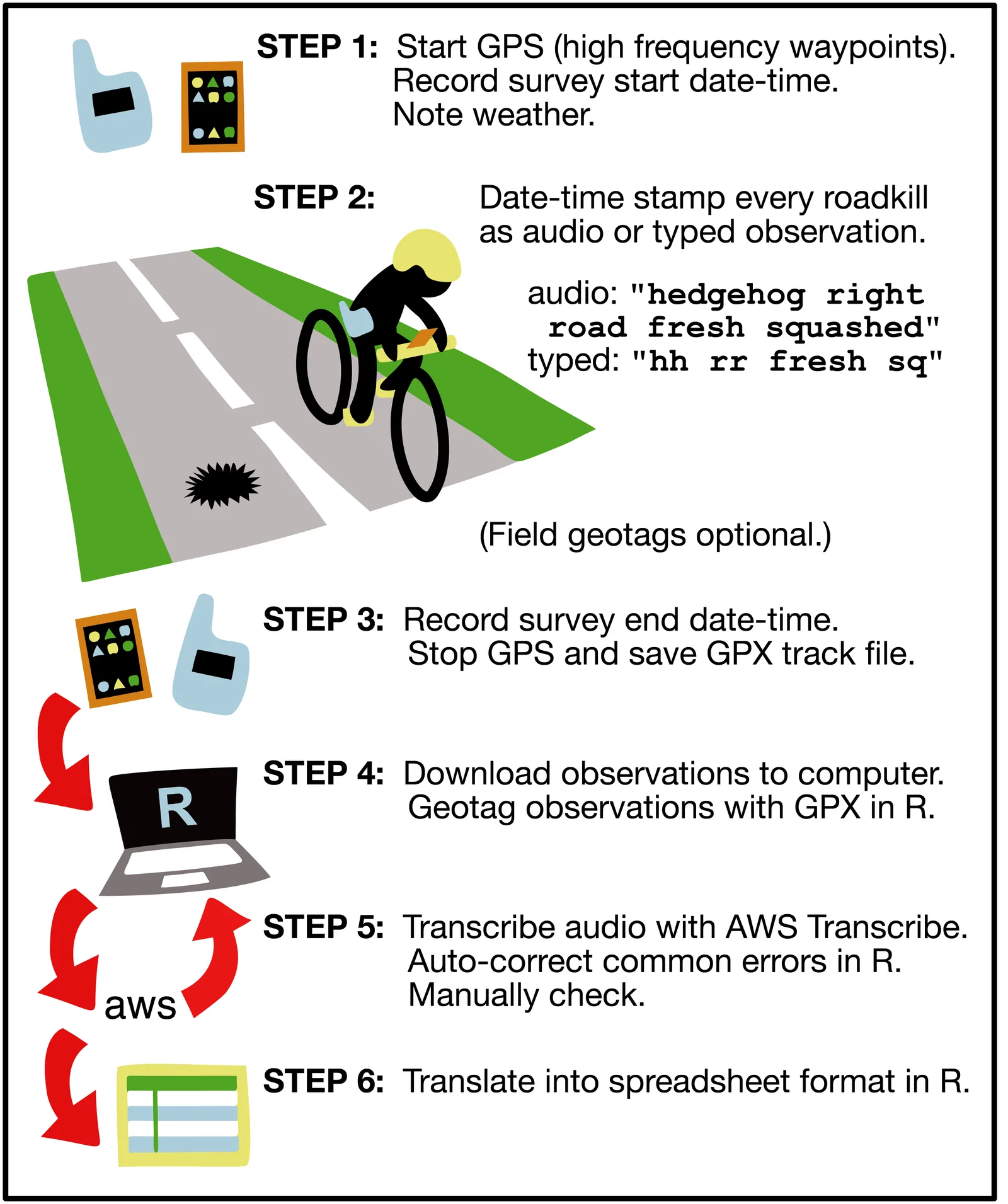
A summary of our road-kill monitoring method. Our use of a smartphone and GPS unit to survey road-kill from a bike. The method can be used from other modes of transport and can use other equipment (for example, the GPS unit can be substituted for a GPS app on a smartphone, and audio notes can be spoken into a smart watch instead of a smartphone). From our experience in New Zealand, individually geotagging each road-kill with a smartphone when on the move is typically less accurate than geotagging date-time stamped observations using a trip GPS Exchange Format (GPX) file with high-frequency waypoints from a GPS unit (or GPS app). That may vary about the world based on GPS coverage. We process our observations in R using Amazon Web Services Transcribe to transcribe audio notes. This could also be achieved with other professional data apps.

Our method (summarised in Fig. 1) allows for the convenient collection of individually geotagged road-kill while moving on foot, by bicycle, or as a passenger in a motor vehicle. It is designed to quickly map out all road-kill visible along survey routes. (Note that portions of this text were previously published as part of a preprint ‘https://www.biorxiv.org/content/10.1101/2025.05.06.652546v1.full’.) Dr. Jon Sullivan, Lincoln University, has used this method, or precursors of it, to collect almost daily road-kill data beginning in 2003, mainly around Ōtautahu-Christchurch, NZ (see case study below). At a minimum, the method requires a device recording a Global Positioning System (GPS) track and a means of recording timestamped written or audio notes. All can be provided by a smartphone. It also requires an observer skilled enough to identify road-kill to species while moving. While we emphasise here that valuable road-kill data can be collected while moving, this does not preclude an observer from pausing the survey to stop to inspect particularly notable road-kill.

The survey is marked with a start and stop time, and then each road-kill along the route is marked with a timestamped note, either written in shorthand or spoken. This proved to be much faster data entry than more complex interfaces with drop-down options or buttons to browse through. There are many apps that can create timestamped notes. For audio notes, a simple voice recorder can be sufficient, so long as it accurately records the start time of each recording (*e.g.*, Voice Memos on smartphones and smart watches). We use FileMaker Go for its flexibility in recording timestamped (and geotagged) text, audio, and handwritten notes. The TRYVL app for mobile devices is another option that geotags voice memos and the Obsidian app can do this using the community plug-ins tidit and Commander.

The timestamps of all road-kill observations can later be geotagged with the GPS track. We have found that geotagging against a continually recording GPS track provides substantially more accurate coordinates that relying on a smartphone’s point geotags (see also Osborne et al., 2025). Points by a smartphone while moving along a road can lag by many tens of metres. The continuous GPS track can be made by a smartphone app (we use the Cyclemeter app) or using a standalone GPS unit (we use a Garmin GPSMAP 64). An additional benefit of recording a GPS track on each survey is that observer speeds can be calculated throughout the survey.

Road-kill observations can be either written in shorthand or spoken using an analogous syntax. Each uses the following structure, where asterisked elements are compulsory and non-asterisked elements are optional:

[species name]* [identification uncertainty] [position on/by road]* [road-kill age]* [road-kill condition]* ([alternative identifications] [new or usual carcass] [other notes, *e.g.*, sex, life stage, photo])

For example, we could type “hh lr old sq (usual)” or speak “hedgehog left road old squashed usual”. Both indicate that at this location there was a road-kill carcass of a hedgehog (*Erinaceus europaeus*) on the left side of the road that was old (definitely >24 h old) and squashed on the road. “Usual” indicates that it was a carcass that had been already recorded on a previous survey and was still present. We expand on the rationale and categories for each of these elements below.

### Road-kill taxon

Each road-kill observation begins with the taxon name. Typically this is a species name although it can be a higher taxon when the identification is difficult (*e.g.*, raptor, bird, mammal). A user-maintained list matching short-hand names to scientific names is required (*e.g.*, hh for hedgehog, *Erinaceus europaeus*).

When the observer is not 100% certain of a species identification, it is always followed by a question mark (*e.g.*, “hh?” in shorthand, “hedgehog question mark” in spoken audio). In rare cases where it is not certain whether an object on the road is a road-kill, a double question mark is used (*e.g.*, “hh??” in shorthand, “hedgehog question mark question mark” in spoken audio). This most often happens when the road-kill is well-worn and observations are made from a fast-moving car. This is important so that the overall road-kill density per kilometre is not influenced by the observer’s decisions around whether objects that look like well-worn carcasses are or are not dead animals.

### Road-kill age

The age of the road-kill when first encountered is coarsely estimated as “fresh” (definitely < 24 h old), “unsure” (possibly < 24 h old), and “old” (definitely > 24 h old). This helps with calculations of road-kills per day along regularly travelled routes. With some experience, especially along regularly surveyed routes, categorising road-kill to this coarseness of age is usually quite easy. See “Road-kill persistence” below for estimating the subsequent time a carcass remains visible.

### Road-kill condition

The condition of a carcass on the road affects its detection probability and its persistence on the road (*e.g.*, availability to scavengers). This is recorded as “intact”, “exposed” (some internal tissues visible but carcass still fully formed), “squashed”, “decomposed” (carcass visibly flattened down due to decomposition), “fragment” (only part of the tissue of the carcass remains), and “skeleton” (only bones are visible). In shorthand, these are abbreviated as “int”, “exp”, “sq”, “decomp”, “frag”, and “skel” respectively. Additional condition categories “fur”, “feathers”, and “blood” are optionally used although in our surveying only carcasses with tissue present have been consistently recorded. Recording and tracking road-kill condition in this way assists with estimates of detectability and persistence.

### Road-kill position

Like condition, the position of the carcass on or by the road affects its detectability and persistence. Carcasses on the road in the path of traffic are typically more apparent but less persistent. For simplicity, seven categories are used, “mid road” (within 30 cm of the road centre line), “left road” and “right road” (on the road in the path of vehicles, relative to the direction of the observer), “left verge” and “right verge” (either beyond the roadside boundary line but still on the road, or within 30 cm of the road edge away from the path of car tyres), and “left roadside” and “right roadside” (synonymous in our data collection with “left grass” and “right grass”). These categories are abbreviated in shorthand as “mr”, “lr”, “rr”, “lv”, “rv”, “lg”, “rg” respectively. “Left road” and “right road” can be optionally replaced with compass directions for added clarity (“east road”, “west road”, “north road”, “south road”). This can be useful when on foot at complex intersections where “right” and “left” may be misinterpreted. Recording road-kill position assists with determining if a carcass is a new entry or a previously recorded usual carcass, as well as providing nuanced estimates of persistence.

### Road-kill persistence

Understanding road-kill persistence and detectability are both assisted by repeat observations of the same carcasses. In our method, all carcasses are recorded on every survey, regardless of whether or not they have been recorded previously. All previously recorded carcasses are noted with the special word “usual” (with care being taken not to use “usual” in other contexts in notes). When it is uncertain whether or not a carcass is usual, “possibly usual” or “probably usual” are used. For frequently repeated surveys, this knowledge of which carcasses are new and which are usual can be used to provide estimates of road-kill persistence for different taxa, in different conditions, on different positions on and by the road. That in turn can be used to better interpret the road-kill rates from data collected on less frequently travelled routes. On routes that vary in traffic volumes, the frequency with which new and usual carcasses are missed by observers could also allow the effects of traffic on observers’ detection probabilities to be estimated.

### Other notes

Other notes about a carcass are optionally added after the essentials. In shorthand, these notes are in parentheses. In spoken notes, they are just said after the essentials. When apparent, sex and life stage (“adult”, “juvenile”, “baby”) are noted. The word “photo” is exclusively used when a photo of a carcass is taken.

### Multiple carcasses at one location

When there are multiple carcasses at one location, each individual animal is always entered separately. For shorthand notes, this is done by putting each carcass on a separate line of one geotagged note. In spoken audio, each observation is separated by “and also with”. For example,

pd mr fresh int (female)

pd lr fresh sq (baby)

pd rr fresh sq (baby)

“paradise shelduck mid road fresh intact female and also paradise shelduck left road fresh squashed baby and also paradise shelduck right road fresh squashed baby”

This typed shorthand, or equivalent spoken note, indicates that the carcass of an adult female paradise shelduck was in the middle of the road and two paradise shelduck ducklings were squashed, one on the left side of the road and one on the right side.

### Processing audio notes

When passenger in a car, or walking, it is practical to type in road-kill as shorthand. Care needs to be taken with smartphones that their automatic spellcheckers do not override what is typed. Shorthand codes can be added to a smartphone’s dictionary to reduce issues with the spellchecker. When biking and running, speaking audio notes is the easier data entry method. This then creates many audio files that need to be transcribed. While this could potentially be done on device, there is typically not the time while surveying to check and correct on-device transcription results. Instead, we have found that the most efficient workflow is to transcribe the recordings later at a computer.

We use Amazon Web Services Transcribe speech-to-text web service, since it offers many English accent options, it takes a vocabulary file of common spoken words and phrases, and it allows models to be trained on provided data (we update this regularly with all our manually checked data).

The results from AWS Transcribe are good, although not 100% accurate, especially for noisy field recordings. We maintain a database of common transcription errors and use an R script (R; R Foundation for Statistical Computing, Vienna, Austria) to automatically clean the AWS Transcribe results, and then we manually check for remaining errors.

### Data processing

We use Rs with regular expressions to translate the shorthand and spoken observations into spreadsheet format. An example R for expanding the shorthand syntax into an R data.frame is included in the Supplementary Materials. Use of consistent formatting and consistent species names is essential.

### Case study

**Figure 2:**
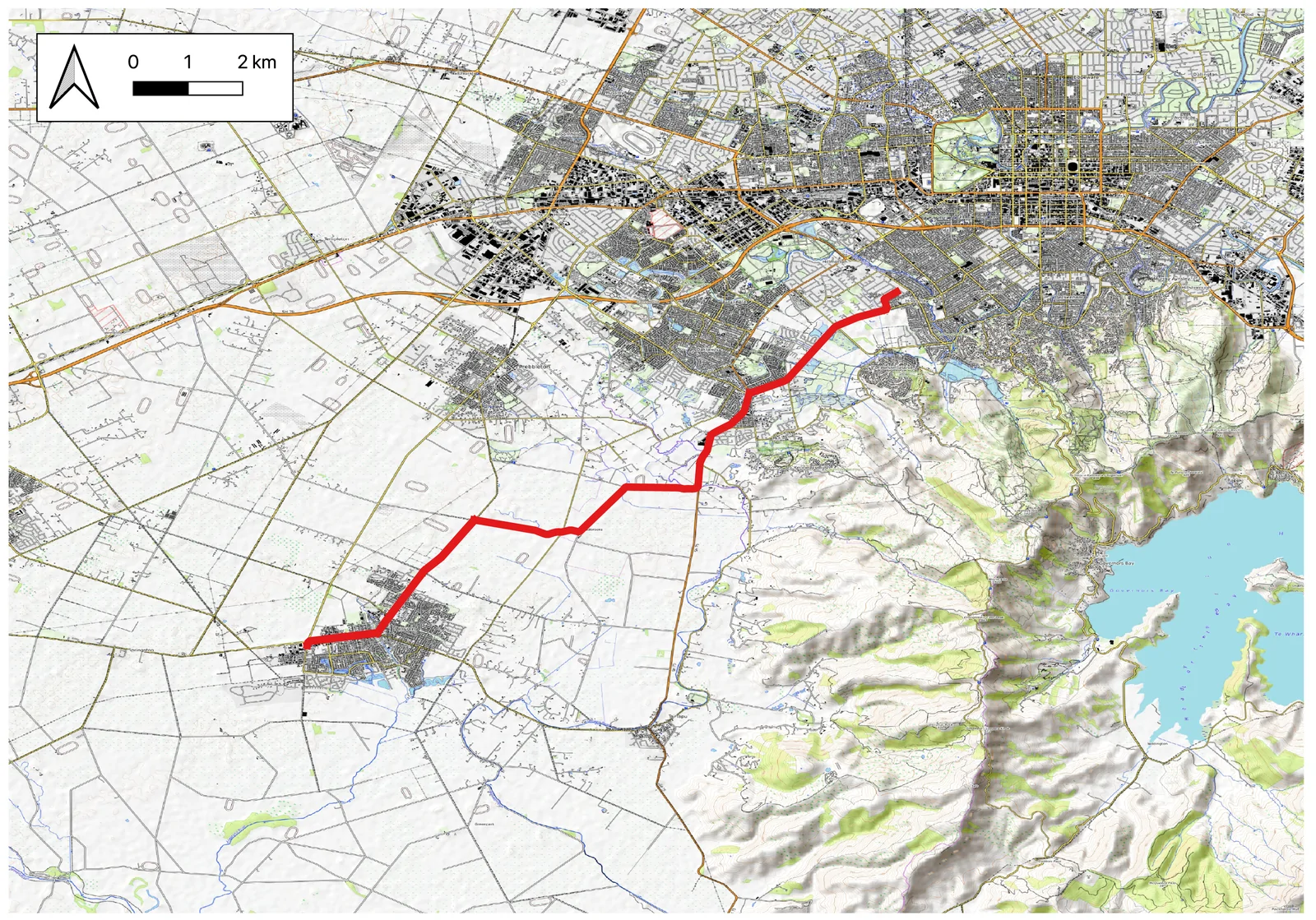
Map showing the 16.2 km road route between Christchurch city and Lincoln town where road-kill data were collected. The area between the suburban housing of Christchurch and Lincoln is primarily pastoral farming. To the southeast are the Port Hills and Banks Peninsula. The background map layer is from Open Topo Map (CC-BY-NC-SA 4.0 OpenTopoMap).

Dr. Jon Sullivan has applied this method to a 16.2 km road route between southwestern Ōtautahi-Christchurch city and Lincoln town in Canterbury, New Zealand (Fig. 2). This route begins in a suburban landscape of mostly single storey dwellings, with speed limits 50–60 km/hr. It then moves through pastoral farmland, with speed limits 80–100 km/hr, before entering the suburban landscape of Lincoln town with a 50 km/hr speed limit. Over the past two decades, some farmland has been converted into housing and traffic density has consequently increased.

Christchurch is a temperate coastal city in the rain shadow of the Southern Alps. Temperature typically ranges from infrequent summer highs over 30 °C to winter night-time lows a few degrees below 0 °C. Average annual rainfall is about 650 mm, spread throughout the year. The built area of the city was originally wetland and evergreen forest on part of the extensive flood plain of the Waimakariri River, which is currently held in place north of the city. Christchurch is now primarily suburban housing and industrial areas surrounded by open pasture and cropping. Many species have been introduced, both on purpose and by accident, almost all since European settlement. Many of the most abundant species in the city are now naturalised introduced species. Many indigenous species are locally, if not nationally, extinct and others are rare.

Along this route our method, or a paper-based precursor of it prior to smartphone apps, has been applied, by a single observer, on 2,745 trips since March 2003, primarily travelling by bicycle and occasionally (<3% of journeys) as a passenger in a car. Surveying this route by bicycle took on average 36 min (26–49 min depending on the wind, s.d. 4.0 min). For the Results section below, we report on the 1,652 road-kill trips since 2 April 2012, which were the data collected using a smartphone with the complete method described here. Over this period, we rode the 16.2 km route on average 1.99 days per week (s.d. 1.43), which varied from 1.15 days per week (s.d. 1.01) in 2015 to 2.81 days per week (s.d. 1.47) in 2023. We focus here on reporting summary statistics that illustrate the usefulness of this method; a detailed analysis of these data, along with data from other routes surveyed in this landscape, will be included in a subsequent manuscript. Since almost all journeys along this route were by bike, it was not appropriate to do a comparison of road-kill rates across different travel methods for this case study. Instead, we include the proportion of new carcasses seen on the road (including road verge) and off the road while on foot, biking, and as a passenger in a car, across our larger database including all routes we have travelled (JJ Sullivan, 2025, unpublished data, 5,757 surveys across many routes). This is a preliminary assessment of how detection of off-road carcasses may decline with travel speed.

The data presented include 59 surveys with typed shorthand and 1,593 surveys with transcribed spoken notes. Of the 35,010 spoken road-kill observations (including repeat observations of the same carcasses on consecutive surveys), 3,496 (10%) were manually checked and corrected if necessary (most of these were observations that our R transcription correcting script flagged as needing checking). The remainder are currently as transcribed by AWS Transcribe then automatically corrected with an R using a database of 13,805 transcription errors (these are errors for both road-kill observations and more broadly for other wild counts made along this route). After this automated error correction, there was a 96.7% per character accuracy in the transcriptions, comparing manually checked observations with pre-checked observations.

The total road-kill rate along this route, in road-kill/100 km/year, was estimated by averaging the per-day number of new road-kill carcasses, divided by 16.2 km then converted to per 100 km, and multiplied by 365. A lower estimate used only the average number of definitely fresh (>24 h old) carcasses detected per day. An upper estimate included the fresh carcasses, possibly fresh (“unsure”) carcasses, and the proportion of old (>24 h old) carcasses that were first detected the day after they were missed.

## Results

### Road-kill taxa

Between 2 April 2012 and 8 March 2024, along one 16.2 km road route, we recorded 4,034 new road-kill carcasses (Table 1). Of the reported road-kill, 62.4% were birds, 31.1% were mammals, and 3% were butterflies. Of the bird road-kill, 18.2% were native species, which reflects the exotic-dominated bird community in this rural and urban landscape. In general, the commonly encountered road-kill were of the most abundant taxa in the surrounding landscape (JJ Sullivan, 2025, unpublished data).

**Table 1:** Summary of road-kill carcasses observed along a 16.4 km road route over a decade. Summary of all road-kill observations recorded in the ten years between 2 April 2012 and 8 March 2024 along one 16.2 km road route. Taxonomic authorities are provided for taxa identified to species level or below. Note that native birds include some recently self- introduced (without human assistance) bird species. All mammalian species in this table are non-native to New Zealand. For the purposes of the survey, the colourful day-flying moth, *Nyctemera annulata*, was consistently included with butterflies. *Note that the mallard and mallard-grey duck hybrids are listed here as an introduced species although grey ducks are native.

**Taxon scientific name**	**Common name**	**New counts**	**Repeat counts**
**All species**		**4,034**	**31,859**
**Birds**		**2,609**	**9,434**
**Native**		**458**	**2,167**
*Anthornis melanura melanura* (Sparrman, 1786)	Korimako/NZ Bellbird	6	3
*Aythya novaeseelandiae* (Gmelin, 1789)	Pāpango/NZ scaup	2	46
*Circus approximans* Peale, 1848	Kāhu/Australasian harrier	29	95
*Cygnus atratus* (Latham, 1790)	Kakīānau/Black swan	3	7
*Gerygone igata* (Quoy & Gaimard, 1830)	Riroriro/grey warbler	11	29
*Haematopus ostralegus finschi* Martens	Tōrea/South Island pied oystercatcher	1	7
*Hemiphaga novaeseelandiae novaeseelandiae * (Gmelin)	Kererū/NZ wood pigeon	1	0
*Himantopus himantopus leucocephalus* Gould, 1837	Poaka/Australasian pied stilt	1	1
*Hirundo neoxena neoxena* Gould, 1842	Warou/welcome swallow	14	15
*Larus dominicanus dominicanus* Lichtenstein, 1823	Karoro/southern black-backed gull	11	19
*Microcarbo melanoleucos brevirostris* (Vieillot, 1817)	Kawaupaka/little pied shag	1	4
*Porphyrio porphyrio melanotus* Temminck	Pūkeko	261	1767
*Rhipidura fuliginosa fuliginosa* (Sparrman, 1787)	Pı¯wakawaka/NZ fantail	31	63
*Tadorna variegata* (Gmelin, 1789)	Pūtangitangi/paradise shelduck	2	1
*Todiramphus sanctus vagans* (Lesson, 1828)	Kōtare/scared kingfisher	1	0
*Vanellus miles novaehollandiae* Stephens, 1819	Spur-winged plover	15	27
*Zosterops lateralis lateralis* (Latham)	Tauhou/silvereye	83	79
**Introduced**		**2,059**	**7,199**
*Acanthis flammea cabaret* (Müller, 1776)	Lesser redpoll	26	42
*Alauda arvensis* Linnaeus, 1758	Eurasian skylark	2	4
*Anas platyrhynchos* Linnaeus, 1758*/A. superciliosa* Gmelin, 1789	Mallard or hybrid with grey duck*	420	3,446
*Athene noctua* (Scopoli, 1769)	Little owl	18	104
*Callipepla californlca* (Shaw, 1789)	California quail	2	4
*Carduelis carduelis britannica* (Hartert)	European goldfinch	133	126
*Carduelis chloris* (Linnaeus, 1758)	European greenfinch	71	130
*Columba livia domestica* Linnaeus, 1766	Rock pigeon	11	70
*Emberiza citrinella* Linnaeus, 1758	Yellowhammer	11	27
*Fringilla coelebs* Linnaeus, 1758	Chaffinch	22	19
*Gallus domesticus domesticus* Linnaeus, 1758	Domestic chicken	4	8
*Gymnorhina tibicen* (Latham, 1801)	Australian magpie	38	631
*Passer domesticus domesticus* Linnaeus, 1758	House sparrow	377	367
*Phasianus colchicus* Linnaeus, 1758	Ring-necked pheasant	12	24
*Prunella modularis* Linnaeus, 1758	Dunnock	11	17
*Sturnus vulgaris* Linnaeus, 1758	Common starling	53	141
*Turdus merula* Linnaeus, 1758	Eurasian blackbird	645	1731
*Turdus philomelos* Brehm, 1831	Song thrush	182	309
	Unidentified bird	92	68
**Mammals**		**1,256**	**22,392**
*Capra hircus* Linnaeus, 1758	Feral goat	2	18
*Dama dama* Linnaeus, 1758	Fallow deer	1	4
*Erinaceus europaeus* Linnaeus, 1758	European hedgehog	731	17,067
*Felis catus* Linnaeus, 1758	Domestic cat	30	272
Lagomorpha	Rabbit, hare	7	1
*Lepus europaeus* Pallas, 1778	European hare	100	668
*Mus musculus* Linnaeus, 1758	House mouse	20	27
*Mustela erminea* Linnaeus, 1758	Stoat	3	37
*Mustela furo* Linnaeus, 1758	Ferret	1	1
*Mustela nivalis vulgaris* Erxleben, 1777	Weasel	14	126
*Oryctolagus cuniculus cuniculus* Linnaeus, 1758	European rabbit	121	705
*Ovis aries*	Sheep	3	38
*Rattus* spp.	Unidentified rat species	34	136
*Rattus norvegicus* (Berkenhout, 1769)	Norway rat	10	2
*Sus scrofa domesticus* (Linnaeus, 1758)	Pig	1	16
*Trichosurus vulpecula* (Kerr, 1792)	Brushtail possum	173	3,275
Mammalia	Unidentified mammal	12	0
**Butterflies**		**125**	**10**
**Native**		**54**	**2**
*Nyctemera annulata* (Boisduval, 1832)	Mōkarakara/magpie moth	20	2
*Vanessa gonerilla gonerilla* (Fabricius, 1775)	Kahukura/NZ red admiral butterfly	20	0
*Vanessa itea* (Fabricius, 1775)	Kahu Kowhai/Yellow admiral butterfly	7	0
*Zizina oxleyi* (Felder & Felder, 1865)	NZ blue butterfly	7	0
**Introduced**		**71**	**8**
*Danaus plexippus* (Linnaeus, 1758)	Monarch butterfly	15	2
*Pieris rapae* (Linnaeus, 1758)	Cabbage white butterfly	56	5
**Other**		**44**	**23**
*Anguilla* spp.	Freshwater eel (native)	1	2
*Litoria* spp.	Bell frog (introduced)	2	8
	Unidentified carcasses	41	13

**Figure 3:**
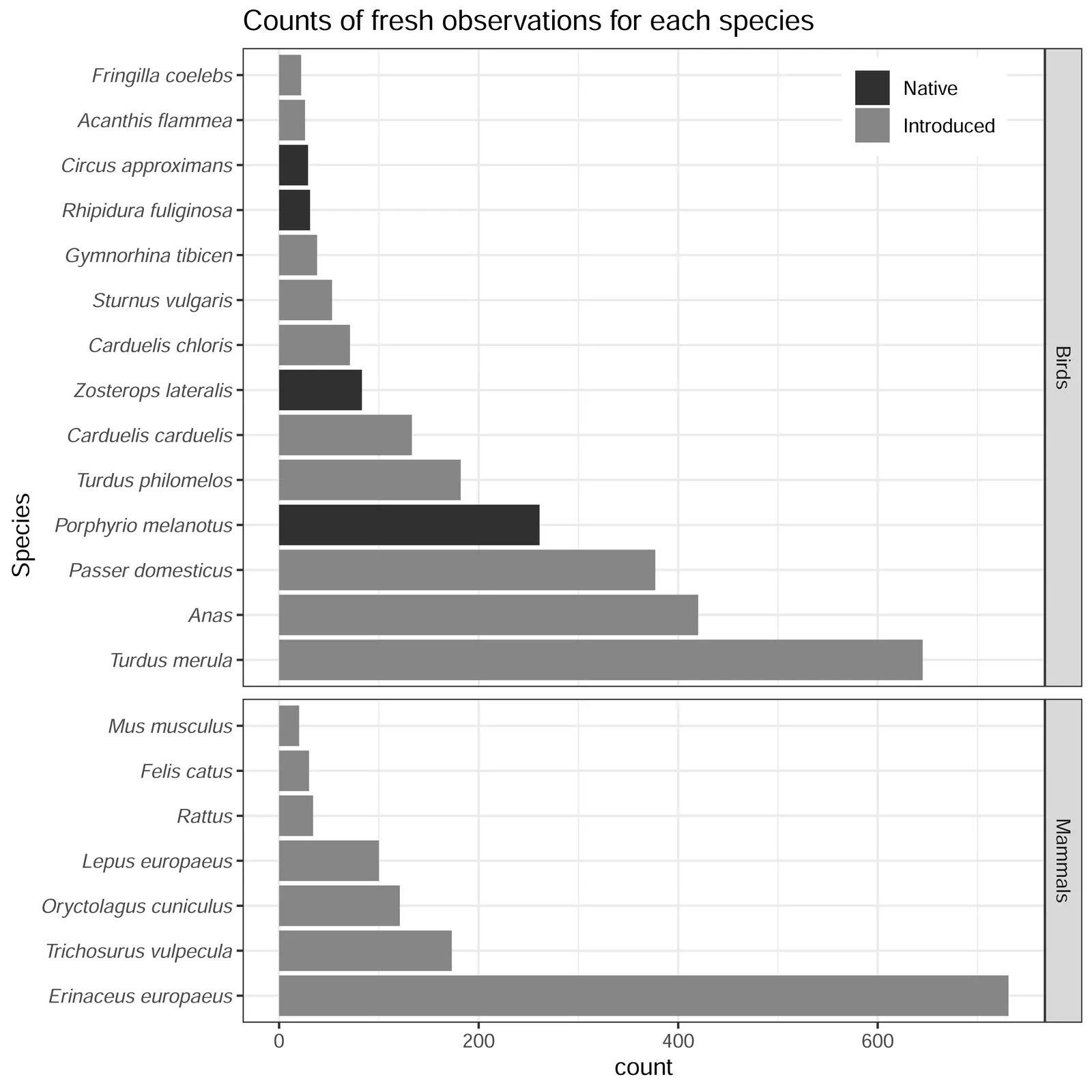
Counts of fresh roadkill observations for each species. Count of fresh carcass (<24 h) observations, for avian and mammalian species with at least 20 fresh carcass observations recorded over the course of 12 years. See Table 1 for the complete taxon names.

Figure 3 shows the numbers of the most commonly recorded species. We plot only fresh carcasses, not newly observed carcasses, to avoid possible bias from the taxon variation in carcass persistence. Figure 3 is therefore an underestimate, unbiased by varying carcass persistence, of the number of each species killed during the survey, and so gives a relative measure of how many of each species were killed. The five most frequently killed species on this road route were, in decreasing order, European hedgehog, Eurasian blackbird, mallard/grey duck (mostly hybrids), house sparrow, and pūkeko (see Table 1 for taxon details).

### Road-kill age

Of the new carcasses, 1,766 (44%) were definitely fresh (<24 h old) at the time of first sighting, while 778 (19%) were possibly fresh (age “unsure”), and the remainder were definitely >24 h old. Of all new road-kill, 873 (20%) were definitely >24 h old and had been missed on the survey the previous day.

### Road-kill condition

Because the route was being surveyed frequently, when road-kill was first reported 17.3% of carcasses were intact and 13.8% had their body cavity exposed but were not yet squashed. Squashed carcasses accounted for 38.3% of newly reported road-kill. As an indication that some new road-kill must have disappeared between surveys and so gone undetected, 22% of road-kill was a fragment of a carcass when first seen, and 1.5% had no body tissue remaining except feathers or fur. Very occasionally black-backed gulls (*Larus dominicanus dominicanus*) and Australasian harriers (*Circus approximans*) were seen removing new carcasses from the road or roadside. Of the repeat reports of old carcasses (“usual” carcasses), 59% were fragments and 21.4% were squashed, with only 3.6% intact and 1.7% exposed.

### Road-kill position

**Figure 4:**
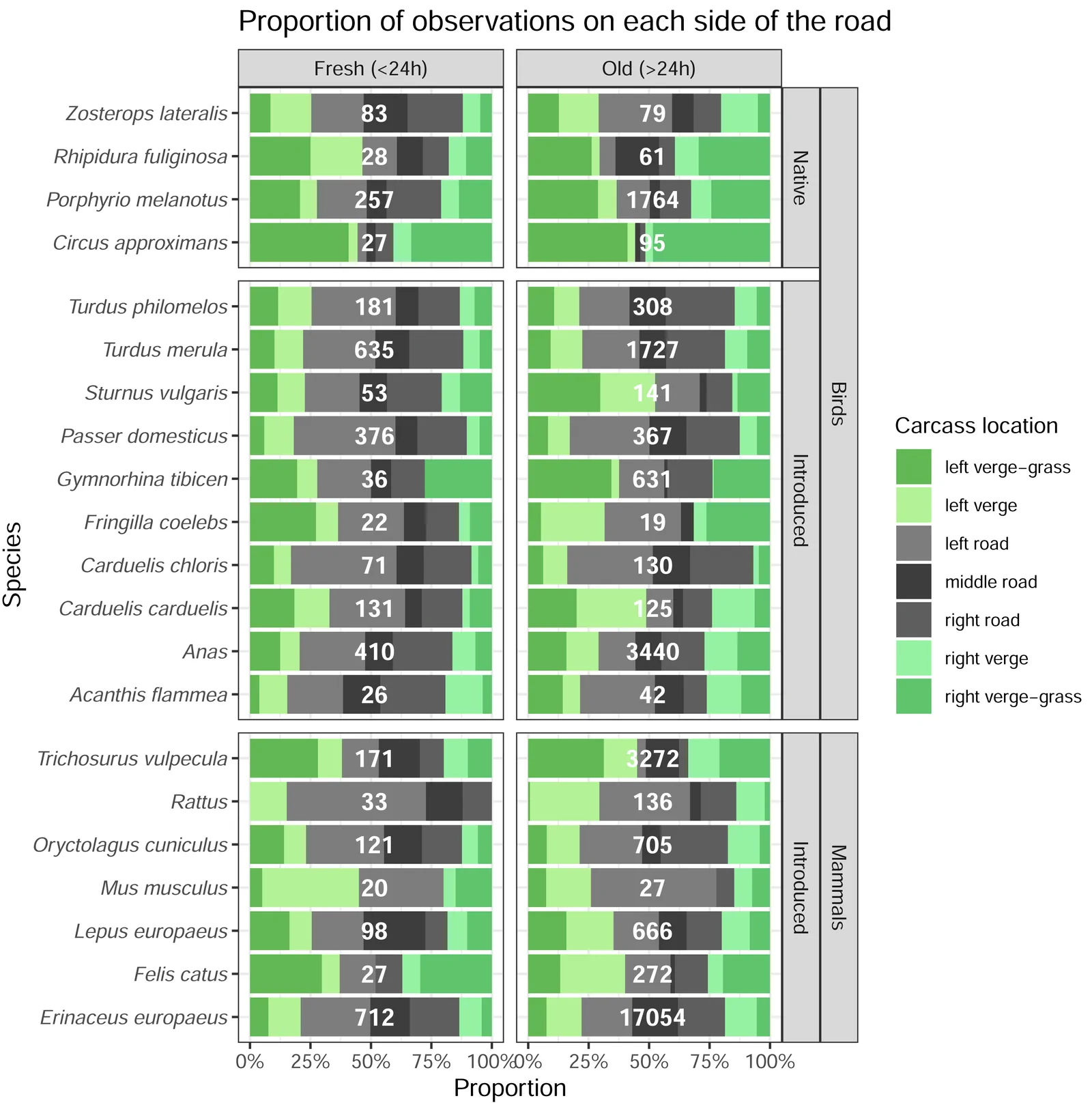
Proportion of roadkill observations on each side of the road. Proportion of carcasses seen on each side of the road, for the avian and mammalian species with at least 20 fresh observations recorded over the course of 12 years. The white labels in the middle of each bar represent the total count of fresh, or old, observations of each species for which a detailed location was recorded.

Of new road-kill, 59% was seen on the road (left-road, mid-road, or right-road), 20% was seen on the paved verge of the road, and 21% was on the land beside the road (“grass” in our shorthand). There was considerable variation among taxa in where on and by the road most road-kill was found (Fig. 4). Australasian harriers, in particular, were almost always seen off the road surface (Fig. 4). These are a large-bodied local scavenger that are typically hit as they fly off the road. In contrast, European hedgehogs were mostly seen squashed onto the road surface, as they are slow-moving and defensively roll up when threatened.

**Figure 5:**
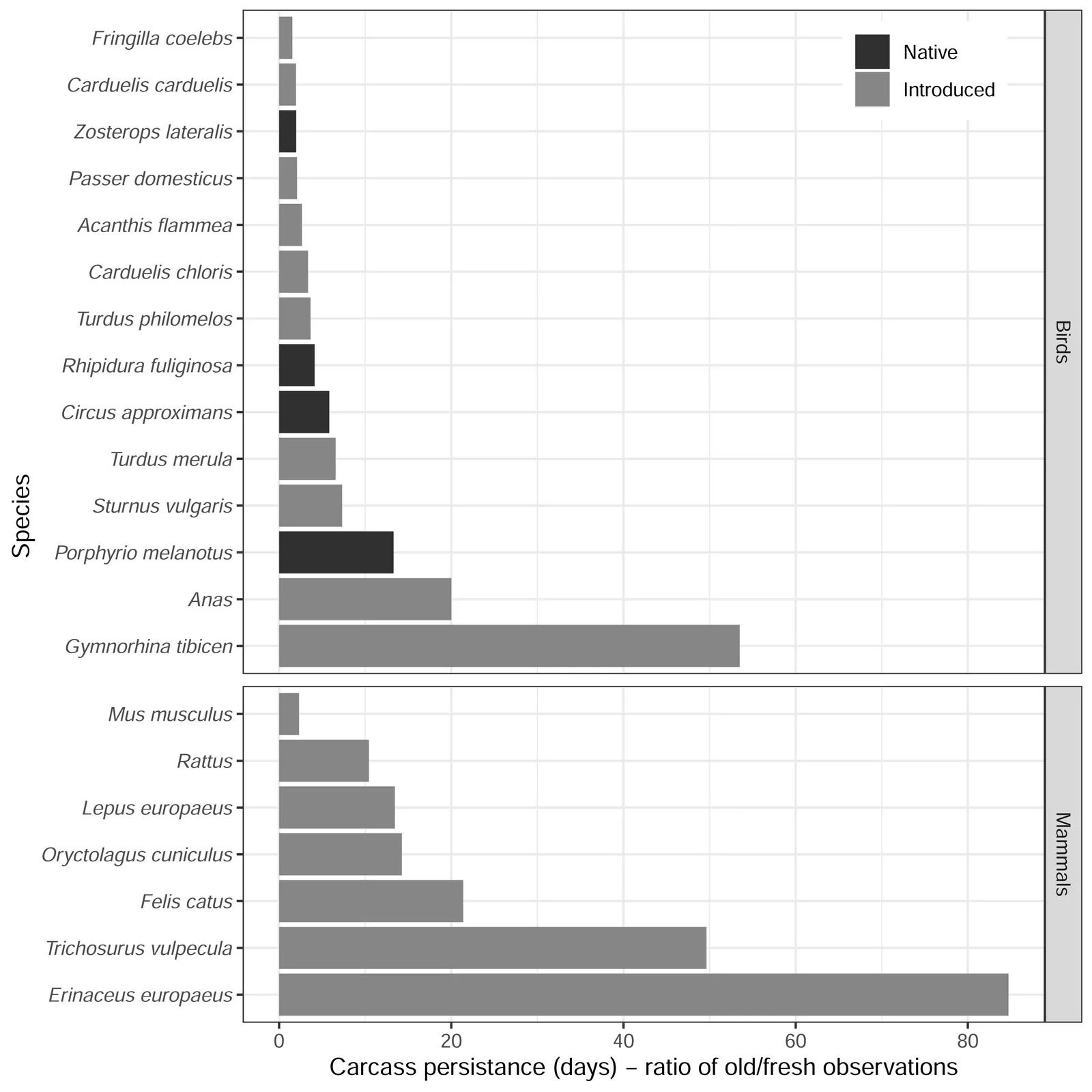
Carcass persistence on the road as estimated by the ratio of old to fresh carcasses for each species. Carcass persistence for avian and mammalian species observed along 16.2 km of road for the avian and mammalian species with at least 20 fresh observations recorded over the course of 12 years. Carcass persistence was calculated as the ratio of old (>24 h) to fresh (<24 h since road-kill) sightings.

Hedgehogs have tough skin and high persistence and so the ratio of carcasses on and by the road varies little between fresh and old carcasses (Fig. 5). In contrast, large birds like pūkeko (*Pophyrio pophyrio melanotus*) and mallards/grey ducks (*Anas platyrhynchos/A. superciliosa*) had lower persistence on the road and older carcasses were disproportionately found off the road where persistence was longer.

Across our larger database, including all road routes travelled (JJ Sullivan, 2025, unpublished data), the percentage of road-kill detected off the road was 26.3% while travelling on foot, 21.1% while travelling by bike, and 8.1% while a passenger in a motor vehicle.

### Road-kill persistence

There were on average 35.0 road-kill recorded per day (s.d. 23.7, *N* = 1025 days), with 11.6% of these being new and the rest being repeated observations of older carcasses. In the landscape surveyed, the combination of frequent re-surveys by the same observer, plus information on the taxon, condition, position, and location, led to individual carcasses being tracked without difficulty. Road-kill of some taxa persisted for many months longer than others (Fig. 5). The longest lasting taxon was the European hedgehog, which on rare occasion lasted on the road for several years (Fig. S2). In comparison, small birds typically lasted for only a few days (Fig. 5). This variation in persistence has a big influence on the road-kill present at any one time. While 66.3% of new carcasses were birds, only 33.2% of all carcasses were birds, due to the longer average persistence of mammal road-kill along this route.

### Road-kill rate

These data can be combined to estimate a total road-kill rate for this section of road. On average there were 1.67 new, fresh road-kill carcasses recorded per day over this 16.2 km route (s.d. = 1.69, *N* = 1,652 surveys), and 0.62 new carcasses per day possibly but not certainly <24 h old (s.d = 1.02). This gives an average estimate of 609.1–835.6 animals killed and detected per year. In addition, 18.1% of carcasses were missed on the first day and first observed when old, which adds a further up to 151.5 animals killed per year. When combined, this equates to 3,760–6,093 road-kill/100 km/year. This will still be an underestimate as it necessarily omits animals that were killed then removed by scavengers between surveys, and those that died away from the road surface in out-of-view locations.

## Discussion

Reliable road-kill data require frequent repeat surveys along the same routes, preferably by the same observers. The method we describe provides a practical way to achieve this consistency. Regular revisits are essential because carcass persistence varies widely among taxa, and infrequent monitoring increases bias by over-representing species whose carcasses remain on the road for longer periods. Repeatedly recording existing carcasses allows persistence to be quantified directly and reduces the uncertainty that arises when carcass turnover is not measured.

We found that carcass persistence can vary considerably among taxa, with tough-skinned taxa like hedgehogs often remaining on the road for many months (and sometimes years) while smaller and weaker skinned taxa would be gone in days to weeks. This needs to be taken into account when interpreting less frequently recorded road-kill data, as taxa with more persistent carcasses will be over-represented in single road-kill surveys. Repeat surveys can also reveal the seasonality of road-kill, and effects of varying weather and traffic volumes on road-kill and its persistence (Erritzoe, Mazgajski & Rejt, 2003; Guinard, Julliard & Barbraud, 2012; Van Der Ree, Smith & Grilo, 2015).

A more complete picture of road-kill is also gained by consistently including the location of each carcass on and by the road. In our example, different taxa were typically found on different parts of the road and road verge (*e.g.*, Australasian harriers were almost always on the grass verge while hedgehogs were almost always on the road surface). Detection probability will generally be higher on the road surface, where carcasses are more visible, than by the sides of the road (something we plan on modelling with our data in a future manuscript). This detection probability interacts with observer speed, with our preliminary assessment showing that observers on foot and bike detected a much greater proportion of road-kill on the roadside than observers in cars. Road-kill persistence also differs based on road location, with carcasses of most taxa persisting considerably longer off the road. These issues mean that road-kill data can contain biases in the taxa detected when road-kill location is not accounted for.

An example of these issues can be found in Brockie, Sadleir & Linklater (2009), New Zealand’s largest published road-kill study to date. The length of New Zealand’s North Island was surveyed for road-kill from a car once a decade for three decades. The majority of the road-kill carcasses in each decade were hedgehogs and brush-tailed possums, with very few small birds seen dead. Our South Island survey data suggests that this result is due to the long persistence of hedgehog and possum carcasses on the road relative to smaller birds (Fig. 5), combined with car-based surveys missing a lot of smaller birds dead in the roadside grass. There is potential to compare road-kill estimates across many published studies once reliable estimates of detectability and persistence have been made for the studied landscapes.

Data from frequent, standardised repeat surveys of road-kill using our method, or similar, will allow per taxon estimates of carcass persistence and detection probability. Using a mix of transport modes along the same route (*e.g.*, walking, running, biking, or travelling as a car passenger) should also make it possible to calculate mode-specific detection probabilities. This is analogous to distance sampling methods (Thomas et al., 2010), where the assumption of perfect detection at distance zero allows estimation of the decline in detection with distance. The equivalent should be achievable for road-kill if carcasses can be assumed to be always detected while walking, with detection declining as observer speed increases. Once persistence and detection probability have been estimated for an area, infrequent surveys such as the per decade survey of Brockie, Sadleir & Linklater (2009) can be calibrated. For example, if the ratio of birds to mammals in our South Island survey (62%) is comparable to the North Island, we estimate that Brockie, Sadleir & Linklater (2009) detected fewer than 9% of bird carcasses. In principle, these repeated-visit data can be analysed using hierarchical detection persistence models, in which each carcass has a latent state process (present or absent) and an observation process (detected or missed). State-space and multi-visit detection frameworks can estimate true carcass persistence and detection probabilities even when carcasses are intermittently missed. We designed the method to generate the structured detection histories required for such models, although formal modelling lies beyond the scope of this methodological study.

Most literature on road-kill has reported on studies that tallied road-kill per unit distance, rather than geotagging individual carcasses. For example, Brockie, Sadleir & Linklater (2009) tallied road-kill to the nearest kilometre using the vehicle’s odometer. Valuable data is lost in this simplification, such as identifying areas of the road, and habitats, that are hotspots for road-kill. Our method demonstrates that this simplification is no longer necessary. In this age of smartphones, it is just as simple for the observer to geotag each carcass when observed. Such data can always be tallied up per unit distance later if needed to compare with past survey results.

Our method separates the concepts of whether a carcass is a new or repeat observation, from the carcass age at detection (<24 h old, uncertain, or >24 h old). We see value in both. Consistently recording whether a carcass is new, or a repeat of a previously recorded carcass (“usual” carcasses), is essential for documenting carcass persistence. In our case, where one observer is traveling the same route almost daily, observer memory, road-kill taxon, condition and position, and a GPS are adequate for tracking which carcasses are new and which are usual. When survey frequency is reduced, and/or multiple observers are used, it may be beneficial to stop and mark carcasses directly. However, doing so will add time and complexity to a survey and we anticipate that in most cases a taxon identification and GPS coordinate should be sufficient to know whether a carcass is new or usual. This repeated recording of the same carcasses also has potential for estimating detection probability, by assessing how many repeat carcasses are missed in surveys.

Estimating carcass age is helping us to assess detection probability (such as when a clearly old carcass is detected for the first time on a route that was recently surveyed, indicating the carcass was previously missed). When combined with carcass persistence, we also expect this will help us to estimate how many carcasses may be killed then removed by scavengers before we record them. For less frequently surveyed routes, this ratio of fresh to old carcasses can be valuable for calibrating biases due to taxon-variable carcass persistence.

The overall road-kill rate in our case study is estimated at 3,760–6,093 road-kill/100 km/year. This is near the high end of international estimates (Calvert et al., 2013) and higher than previous results from previously published New Zealand road-kill studies (Brockie, Sadleir & Linklater, 2009; Sadleir & Linklater, 2016; Flux, Tryjanowski & Zduniak, 2022). The estimates collated by Calvert et al. (2013) ranged from 487 to 37,280 road-kill/100 km/year with a median of 1,488 road-kill/100 km/year. Our estimate, while higher than this median, will be an underestimate as some carcasses will have been missed or removed by scavengers before they could be detected. The combination of surveying by bike plus repeated surveys of carcasses likely make our estimate closer to the true rate than from car-based and less frequently travelled surveys. Collectively, these studies emphasise the role of road-kill as a potentially important source of mortality for species occurring in landscapes characterised by dense road infrastructure and high vehicle speeds.

## Conclusions

The monitoring method described here provides a useful step toward standardised data collection on wildlife mortality along roads. Its versatility and adaptability make it suitable across a wide range of landscapes, habitats, road types, and species, offering broad utility for researchers and conservation practitioners. Relying on readily available technologies such as GPS enabled smartphones and speech to text functionalities, the method allows efficient and accurate recording of carcass locations and associated metadata without requiring observers to stop. Specifically, it captures precise location data through continuous GPS tracking and detailed metadata through speech to text, enabling rapid data collection during regular travel by walking, cycling, or as a passenger in a motor vehicle. Although the method is manual, it uses accessible technologies in ways that reduce cost and increase flexibility, making it efficient and easy to incorporate into daily routines.

While our case study focused on a specific landscape and survey context, the method can be readily adapted for other scenarios. It may be applied to different regions, habitats, or target species, and tailored to address specific research or conservation questions. For example, if temporal variation is a key factor but data on carcass persistence are already available, the method could be modified to sample less frequently and instead focus on covering greater spatial extent or finer seasonal resolution. This approach may be particularly effective for larger species, such as kiwi or penguins, which are likely more easily detected and persist longer on the road surface than passerines. The method also allows for incorporation of updated procedures or new technologies as they become available, ensuring continued relevance in future applications.

Data generated through this approach can support analyses at multiple ecological scales and help reveal patterns in carcass persistence and detection probability, both of which are essential for understanding road-kill impacts. The comprehensive collection of georeferenced data enables identification of road-kill hotspots and provides valuable baseline information for assessing the effectiveness of mitigation efforts. From our experience using this method, it is clear that road-kill detection probability from a moving car is considerably lower than walking or biking, especially for small-bodied taxa and carcasses lying off the road surface. It is important when surveying road-kill to select a means of transport that is suited to the conditions and fauna of the site. While road-kill surveys from moving cars are suitable for some larger-bodied taxa, they are likely insufficient for estimating total road-kill, and car-only road-kill data should be interpreted with caution. Our case study highlights the importance of mixing car observations of road-kill with higher detection probability data from walking and biking in the same landscape, and ideally along the same routes.

By establishing consistent data collection parameters, this method addresses several important sources of bias that have limited previous road-kill research. This improves the comparability and reliability of data across studies. The approach outlined here offers a practical and scalable model for systematic monitoring of wildlife mortality on roads, supporting targeted conservation measures, assessments of population level impacts, and informed mitigation planning within New Zealand and in other regions worldwide.

##  Supplemental Information

10.7717/peerj.21636/supp-1 Supplemental Information 1 Counts of fresh road-kill observations for each species, for all species Count of fresh carcass (<24 h) observations, for all avian and mammalian species with fresh carcass observations recorded over the course of 12 years.

10.7717/peerj.21636/supp-2 Supplemental Information 2 Examples of unusually persistent European hedgehog carcasses observed along the study route (A) An 11 month old carcass on a busy suburban road in Halswell, Christchurch city (speed limit 50 km/hr), first observed on 16 September 2009 and photographed here on 25 August 2010. (B) The remains of a carcass still visible on the road more than 5 years after it was killed, on Robinsons Road, a well-travelled rural road (speed limit 80 km/hr) between Lincoln and Christchurch. This was photographed on 29 April 2024. (C) A carcass 3 months old, also on Robinsons Road, first observed fresh on 12 January 2024 and photographed on 29 April 2024. (D) The same carcass as in (C), here over 22 months old, photographed on 28 November 2025.

10.7717/peerj.21636/supp-3 Supplemental Information 3 Data and R scripts The R script makes the data figures (not Figs. 2 and 2) and table in the manuscript and the summary statistics in the text of the manuscript. The file “data-readme.txt” describes data and explains the columns in the data.

## Additional Information and Declarations

### Data Availability

The following information was supplied regarding data availability: The data and code are available in the Supplemental Files.
