# Supplementary material for: A versatile survey method for repeatedly monitoring individual road-kill
Source: PeerJ. 2026 Sep 21;14:e21636. doi: 10.7717/peerj.21636 (PMC13603908; doi:10.7717/peerj.21636)
Supplement: Supplementary file 1 [file peerj-14-21636-s001.pdf]

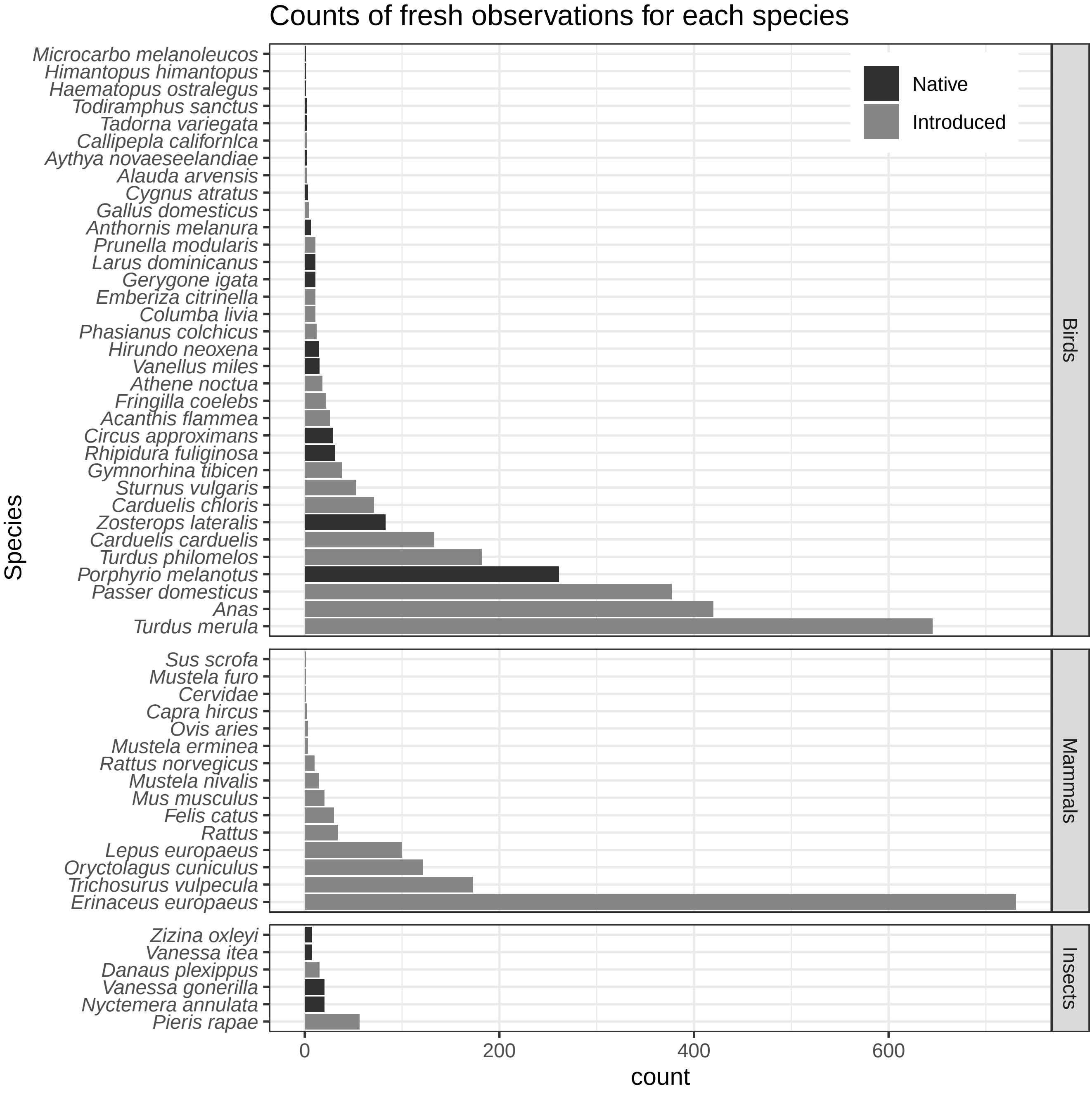

Counts of fresh observations for each species
Native
Introduced
Birds
Mammals
Insects
Species
count
0
200
400
600
Microcarbo melanoleucos
Himantopus himantopus
Haematopus ostralegus
Todiramphus sanctus
Tadorna variegata
Callipepla californlca
Aythya novaeseelandiae
Alauda arvensis
Cygnus atratus
Gallus domesticus
Anthornis melanura
Prunella modularis
Larus dominicanus
Gerygone igata
Emberiza citrinella
Columba livia
Phasianus colchicus
Hirundo neoxena
Vanellus miles
Athene noctua
Fringilla coelebs
Acanthis flammea
Circus approximans
Rhipidura fuliginosa
Gymnorhina tibicen
Sturnus vulgaris
Carduelis chloris
Zosterops lateralis
Carduelis carduelis
Turdus philomelos
Porphyrio melanotus
Passer domesticus
Anas
Turdus merula
Sus scrofa
Mustela furo
Cervidae
Capra hircus
Ovis aries
Mustela erminea
Rattus norvegicus
Mustela nivalis
Mus musculus
Felis catus
Rattus
Lepus europaeus
Oryctolagus cuniculus
Trichosurus vulpecula
Erinaceus europaeus
Zizina oxleyi
Vanessa itea
Danaus plexippus
Vanessa gonerilla
Nyctemera annulata
Pieris rapae
