# Supplementary material for: A versatile survey method for repeatedly monitoring individual road-kill
Source: PeerJ. 2026 Sep 21;14:e21636. doi: 10.7717/peerj.21636 (PMC13603908; doi:10.7717/peerj.21636)
Supplement: Supplementary file 2 [file peerj-14-21636-s002.pdf]

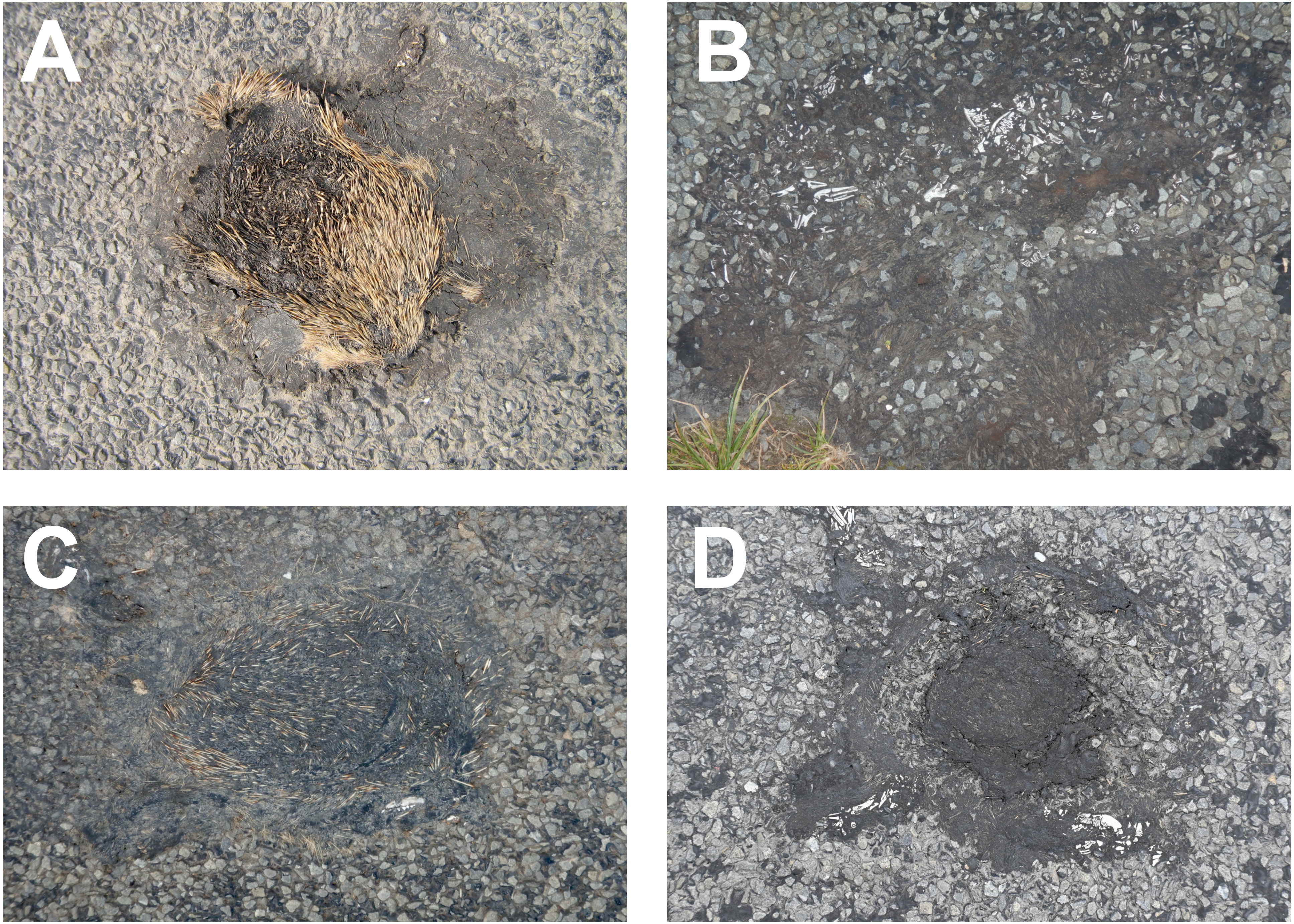
A
B
C
D
