# Supplementary material for: A versatile survey method for repeatedly monitoring individual road-kill
Source: PeerJ. 2026 Sep 21;14:e21636. doi: 10.7717/peerj.21636 (PMC13603908; doi:10.7717/peerj.21636)
Supplement: Supplementary file 3 [file peerj-14-21636-s003.zip › road data and analysis for publication/2-figures&tables/Figure2.pdf]

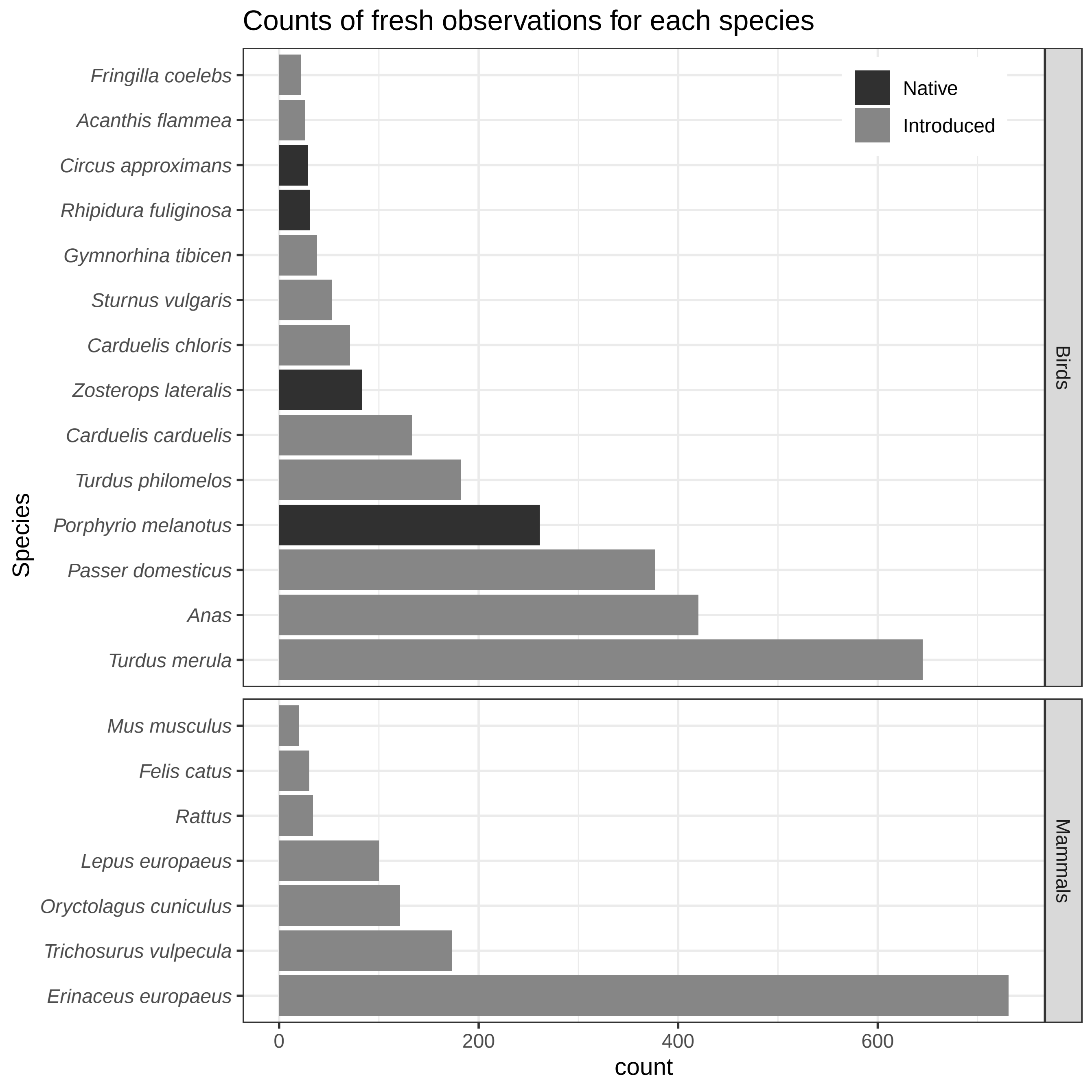

Counts of fresh observations for each species
Native
Introduced
Fringilla coelebs
Acanthis flammea
Circus approximans
Rhipidura fuliginosa
Gymnorhina tibicen
Sturnus vulgaris
Carduelis chloris
Zosterops lateralis
Carduelis carduelis
Turdus philomelos
Porphyrio melanotus
Passer domesticus
Anas
Turdus merula
Birds
Mus musculus
Felis catus
Rattus
Lepus europaeus
Oryctolagus cuniculus
Trichosurus vulpecula
Erinaceus europaeus
Mammals
Species
0
200
400
600
count
