# Supplementary material for: A versatile survey method for repeatedly monitoring individual road-kill
Source: PeerJ. 2026 Sep 21;14:e21636. doi: 10.7717/peerj.21636 (PMC13603908; doi:10.7717/peerj.21636)
Supplement: Supplementary file 3 [file peerj-14-21636-s003.zip › road data and analysis for publication/2-figures&tables/Figure3.pdf]

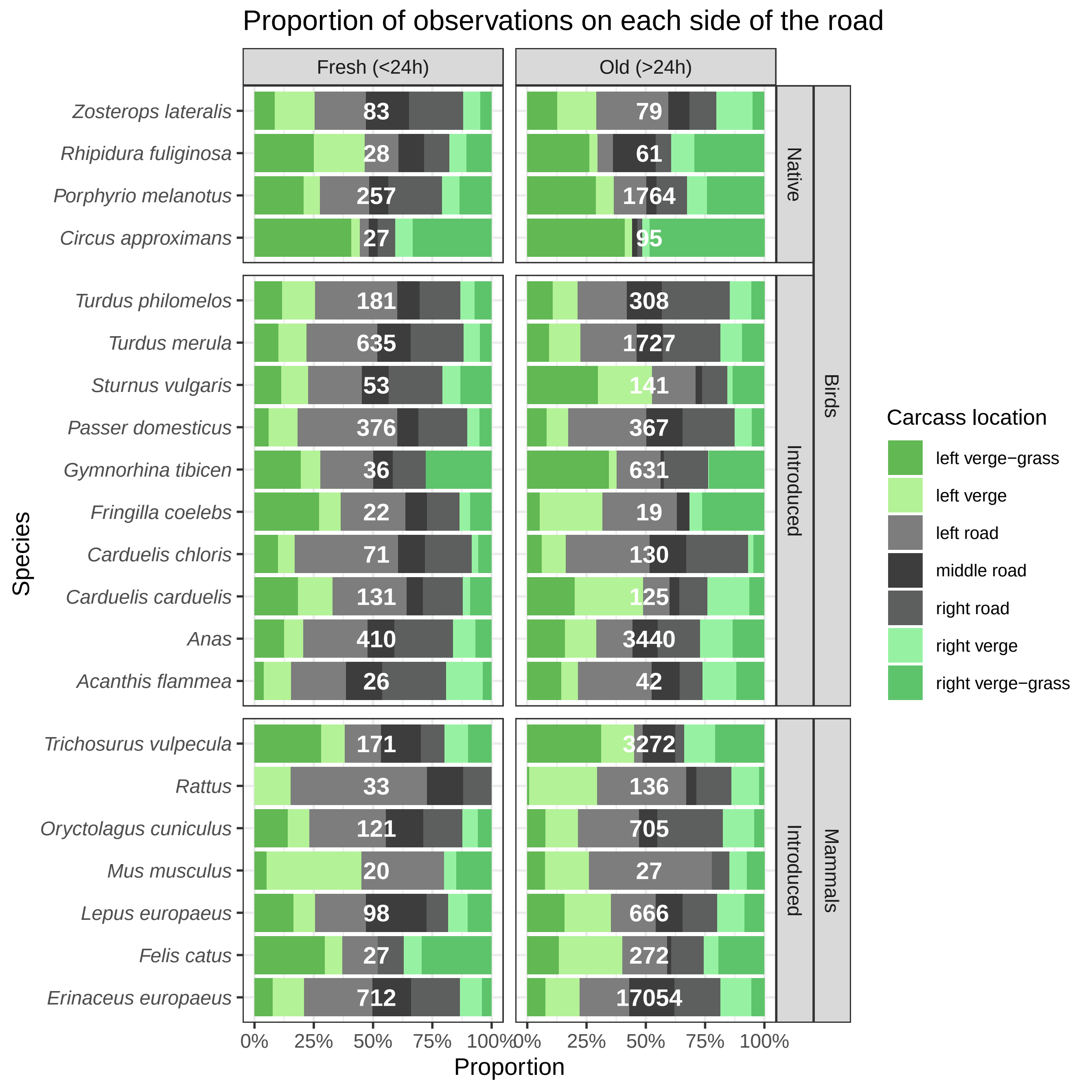

Proportion of observations on each side of the road
Fresh (<24h)
Old (>24h)
Zosterops lateralis
Rhipidura fuliginosa
Porphyrio melanotus
Circus approximans
Turdus philomelos
Turdus merula
Sturnus vulgaris
Passer domesticus
Gymnorhina tibicen
Fringilla coelebs
Carduelis chloris
Carduelis carduelis
Anas
Acanthis flammea
Trichosurus vulpecula
Rattus
Oryctolagus cuniculus
Mus musculus
Lepus europaeus
Felis catus
Erinaceus europaeus
83
28
257
27
181
635
53
376
36
22
71
131
410
26
171
33
121
20
98
27
712
79
61
1764
95
308
1727
141
367
631
19
130
125
3440
42
3272
136
705
27
666
272
17054
Native
Introduced
Birds
Mammals
Species
0%
25%
50%
75%
100%
Proportion
Carcass location
left verge−grass
left verge
left road
middle road
right road
right verge
right verge−grass
