# Supplementary material for: A versatile survey method for repeatedly monitoring individual road-kill
Source: PeerJ. 2026 Sep 21;14:e21636. doi: 10.7717/peerj.21636 (PMC13603908; doi:10.7717/peerj.21636)
Supplement: Supplementary file 3 [file peerj-14-21636-s003.zip › road data and analysis for publication/2-figures&tables/Figure4.pdf]

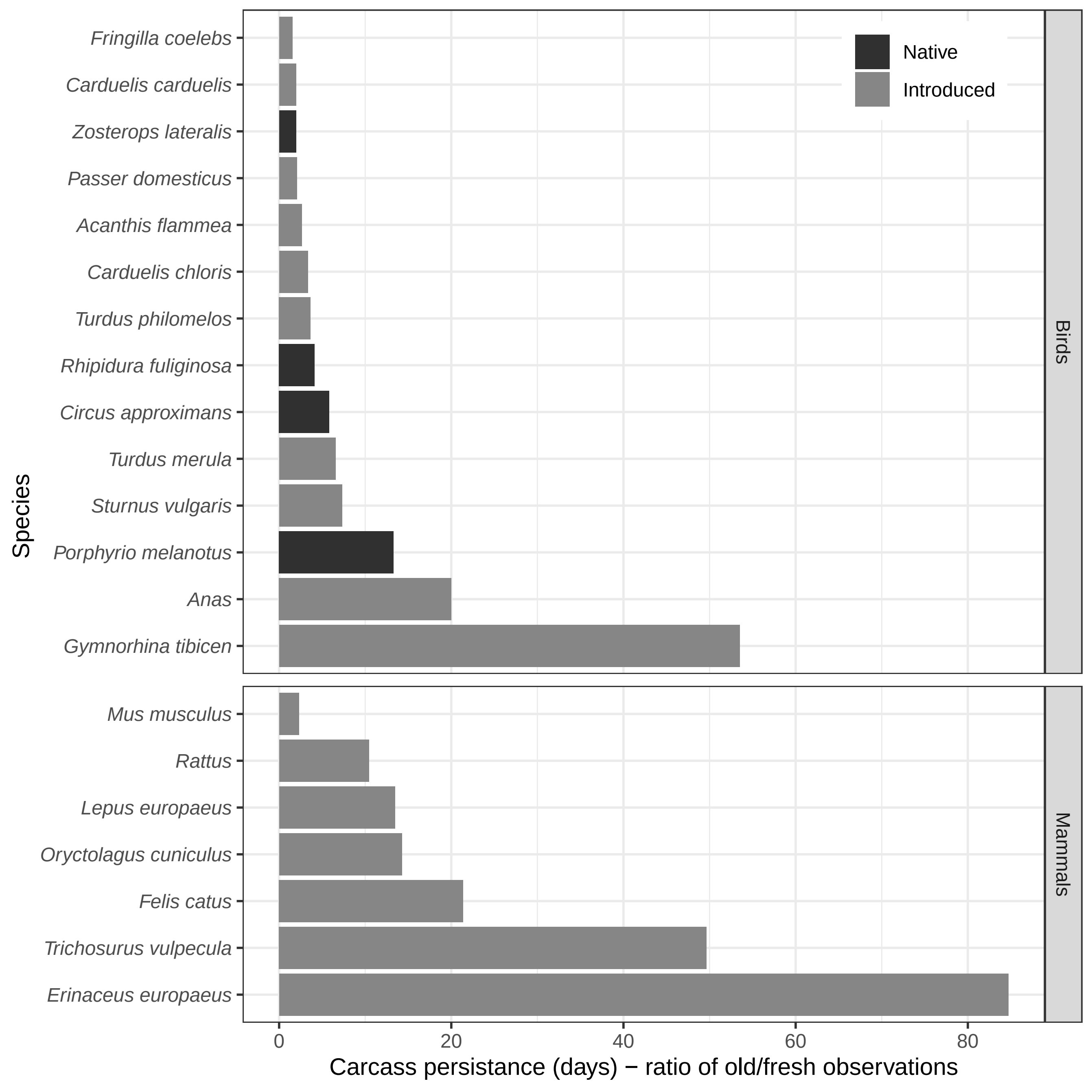

Native
Introduced
Birds
Mammals
Species
Fringilla coelebs
Carduelis carduelis
Zosterops lateralis
Passer domesticus
Acanthis flammea
Carduelis chloris
Turdus philomelos
Rhipidura fuliginosa
Circus approximans
Turdus merula
Sturnus vulgaris
Porphyrio melanotus
Anas
Gymnorhina tibicen
Mus musculus
Rattus
Lepus europaeus
Oryctolagus cuniculus
Felis catus
Trichosurus vulpecula
Erinaceus europaeus
0
20
40
60
80
Carcass persistance (days) − ratio of old/fresh observations
